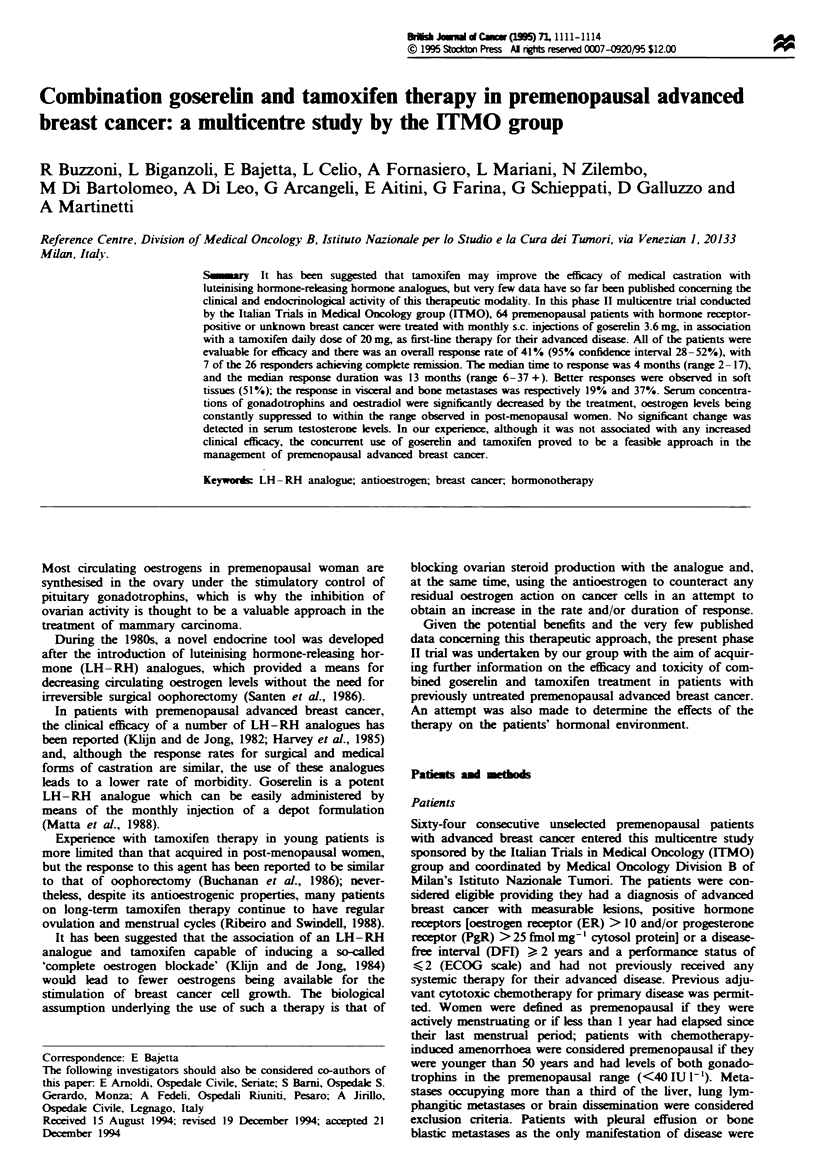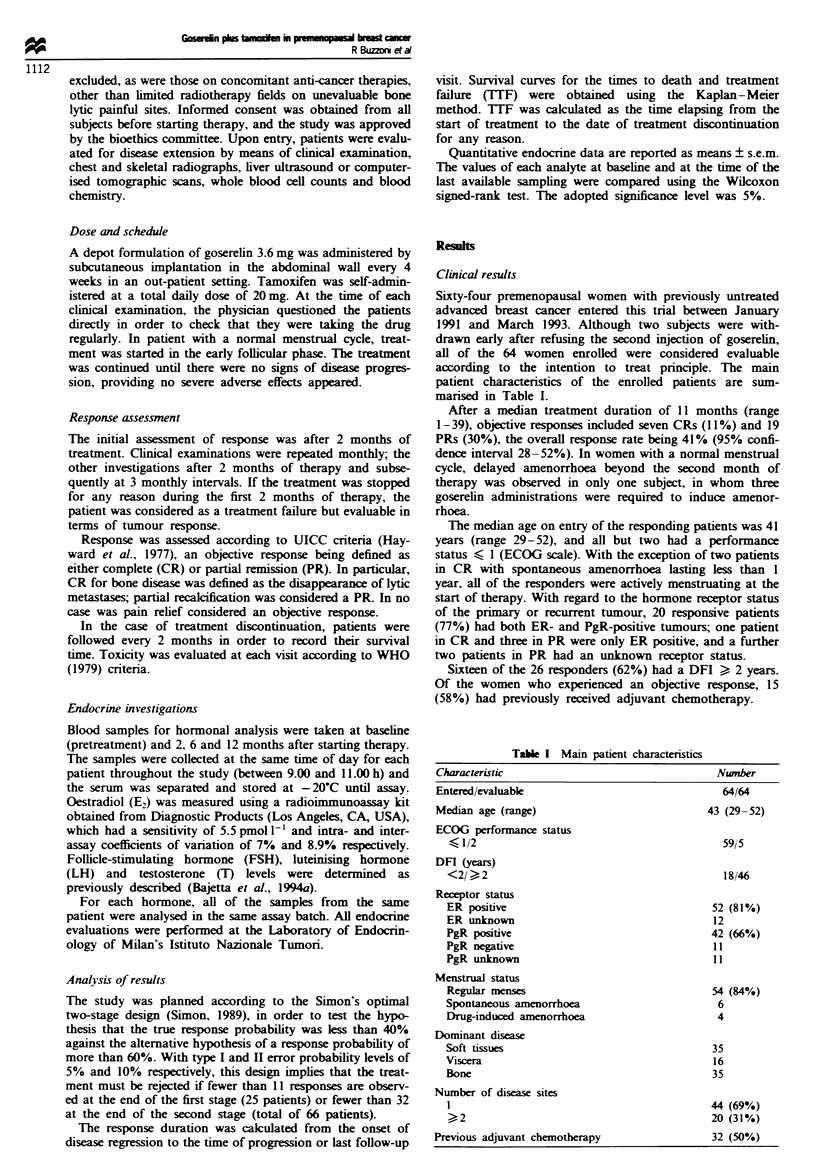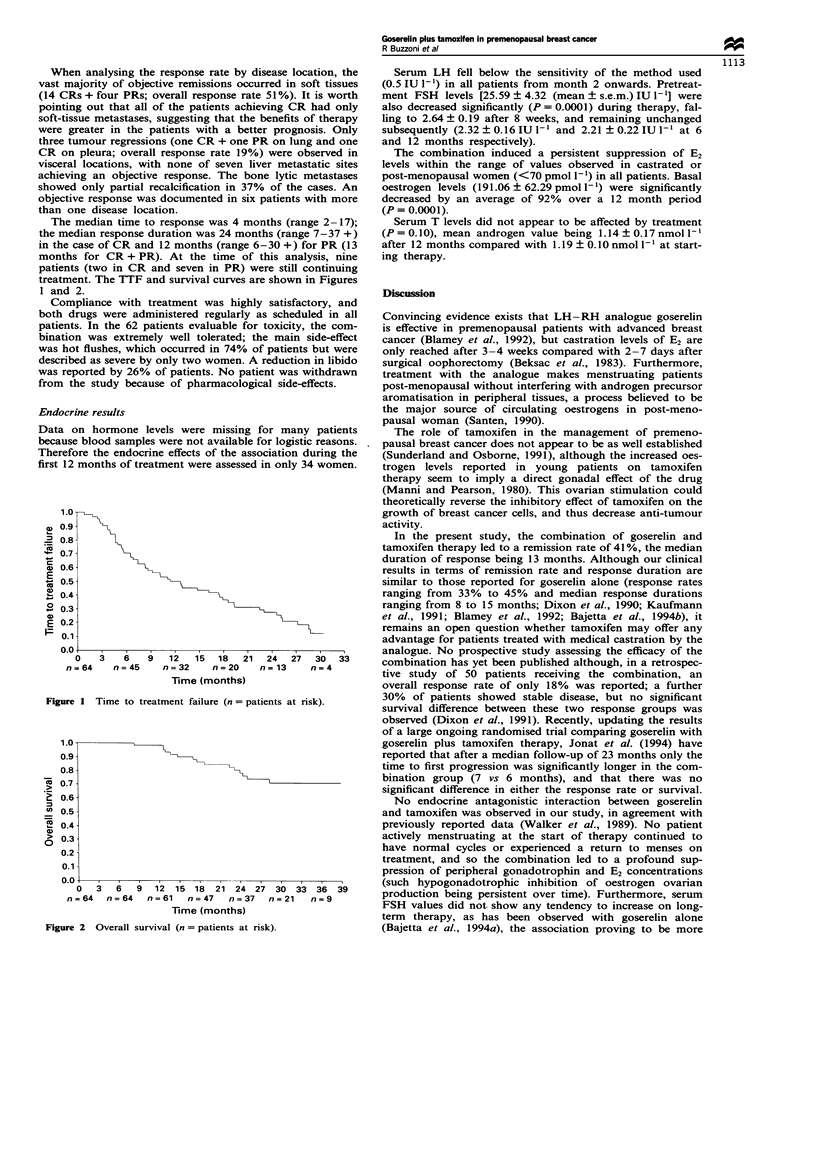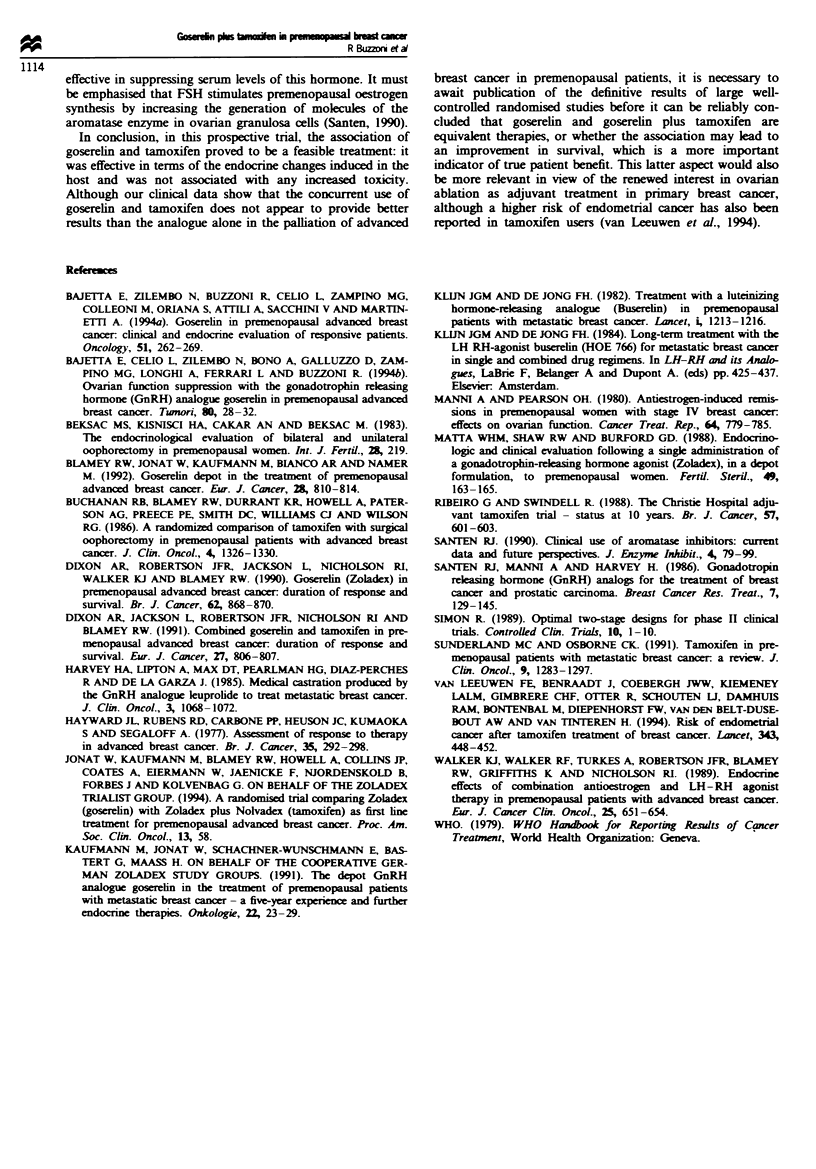# Combination goserelin and tamoxifen therapy in premenopausal advanced breast cancer: a multicentre study by the ITMO group. Italian Trials in Medical Oncology.

**DOI:** 10.1038/bjc.1995.215

**Published:** 1995-05

**Authors:** R. Buzzoni, L. Biganzoli, E. Bajetta, L. Celio, A. Fornasiero, L. Mariani, N. Zilembo, M. Di Bartolomeo, A. Di Leo, G. Arcangeli

**Affiliations:** Reference Centre, Istituto Nazionale per lo Studio e la Cura dei Tumori, Italy.

## Abstract

It has been suggested that tamoxifen may improve the efficacy of medical castration with luteinising hormone-releasing hormone analogues, but very few data have so far been published concerning the clinical and endocrinological activity of this therapeutic modality. In this phase II multicentre trial conducted by the Italian Trials in Medical Oncology group (ITMO), 64 premenopausal patients with hormone receptor-positive or unknown breast cancer were treated with monthly s.c. injections of goserelin 3.6 mg, in association with a tamoxifen daily dose of 20 mg, as first-line therapy for their advanced disease. All of the patients were evaluable for efficacy and there was an overall response rate of 41% (95% confidence interval 28-52%), with 7 of the 26 responders achieving complete remission. The median time to response was 4 months (range 2-17), and the median response duration was 13 months (range 6-37 +). Better responses were observed in soft tissues (51%); the response in visceral and bone metastases was respectively 19% and 37%. Serum concentrations of gonadotrophins and oestradiol were significantly decreased by the treatment, oestrogen levels being constantly suppressed to within the range observed in post-menopausal women. No significant change was detected in serum testosterone levels. In our experience, although it was not associated with any increased clinical efficacy, the concurrent use of goserelin and tamoxifen proved to be a feasible approach in the management of premenopausal advanced breast cancer.


© 1995 Stockton Press All rights reserved 0007-0920/95 $12.00

### Combination goserelin and tamoxifen therapy in premenopausal advanced breast cancer: a multicentre study by the ITMO group

R Buzzoni, L Biganzoli, E Bajetta, L Celio, A Fornasiero, L Mariani, N Zilembo, M Di Bartolomeo, A Di Leo, G Arcangeli, E Aitini, G Farina, G Schieppati, D Galluzzo and A Martinetti

*Reference Centre, Division of Medical Oncology B, Istituto Nazionale per lo Studio e la Cura dei Tumori, via Venezian 1, 20133 Milan, Italy.*

**Summary** It has been suggested that tamoxifen may improve the efficacy of medical castration with luteinising hormone-releasing hormone analogues, but very few data have so far been published concerning the clinical and endocrinological activity of this therapeutic modality. In this phase II multicentre trial conducted by the Italian Trials in Medical Oncology group (ITMO), 64 premenopausal patients with hormone receptor-positive or unknown breast cancer were treated with monthly s.c. injections of goserelin 3.6 mg, in association with a tamoxifen daily dose of 20 mg, as first-line therapy for their advanced disease. All of the patients were evaluable for efficacy and there was an overall response rate of 41% (95% confidence interval 28-52%), with 7 of the 26 responders achieving complete remission. The median time to response was 4 months (range 2-17), and the median response duration was 13 months (range 6-37+). Better responses were observed in soft tissues (51%); the response in visceral and bone metastases was respectively 19% and 37%. Serum concentrations of gonadotrophins and oestradiol were significantly decreased by the treatment, oestrogen levels being constantly suppressed to within the range observed in post-menopausal women. No significant change was detected in serum testosterone levels. In our experience, although it was not associated with any increased clinical efficacy, the concurrent use of goserelin and tamoxifen proved to be a feasible approach in the management of premenopausal advanced breast cancer.

**Keywords:** LH-RH analogue; antioestrogen; breast cancer; hormonotherapy

Most circulating oestrogens in premenopausal woman are synthesised in the ovary under the stimulatory control of pituitary gonadotrophins, which is why the inhibition of ovarian activity is thought to be a valuable approach in the treatment of mammary carcinoma.

During the 1980s, a novel endocrine tool was developed after the introduction of luteinising hormone-releasing hormone (LH-RH) analogues, which provided a means for decreasing circulating oestrogen levels without the need for irreversible surgical oophorectomy (Santen *et al.*, 1986).

In patients with premenopausal advanced breast cancer, the clinical efficacy of a number of LH-RH analogues has been reported (Klijn and de Jong, 1982; Harvey *et al.*, 1985) and, although the response rates for surgical and medical forms of castration are similar, the use of these analogues leads to a lower rate of morbidity. Goserelin is a potent LH-RH analogue which can be easily administered by means of the monthly injection of a depot formulation (Matta *et al.*, 1988).

Experience with tamoxifen therapy in young patients is more limited than that acquired in post-menopausal women, but the response to this agent has been reported to be similar to that of oophorectomy (Buchanan *et al.*, 1986); nevertheless, despite its antioestrogenic properties, many patients on long-term tamoxifen therapy continue to have regular ovulation and menstrual cycles (Ribeiro and Swindell, 1988).

It has been suggested that the association of an LH-RH analogue and tamoxifen capable of inducing a so-called 'complete oestrogen blockade' (Klijn and de Jong, 1984) would lead to fewer oestrogens being available for the stimulation of breast cancer cell growth. The biological assumption underlying the use of such a therapy is that of blocking ovarian steroid production with the analogue and, at the same time, using the antioestrogen to counteract any residual oestrogen action on cancer cells in an attempt to obtain an increase in the rate and/or duration of response.

Given the potential benefits and the very few published data concerning this therapeutic approach, the present phase II trial was undertaken by our group with the aim of acquiring further information on the efficacy and toxicity of combined goserelin and tamoxifen treatment in patients with previously untreated premenopausal advanced breast cancer. An attempt was also made to determine the effects of the therapy on the patients' hormonal environment.

### Patients and methods

### *Patients*

Sixty-four consecutive unselected premenopausal patients with advanced breast cancer entered this multicentre study sponsored by the Italian Trials in Medical Oncology (ITMO) group and coordinated by Medical Oncology Division B of Milan's Istituto Nazionale Tumori. The patients were considered eligible providing they had a diagnosis of advanced breast cancer with measurable lesions, positive hormone receptors [oestrogen receptor (ER) $>10$ and/or progesterone receptor (PgR) $>25$ fmol $mg^{-1}$ cytosol protein] or a disease-free interval (DFI) $\geqslant 2$ years and a performance status of $\leqslant 2$ (ECOG scale) and had not previously received any systemic therapy for their advanced disease. Previous adjuvant cytotoxic chemotherapy for primary disease was permitted. Women were defined as premenopausal if they were actively menstruating or if less than 1 year had elapsed since their last menstrual period; patients with chemotherapy-induced amenorrhoea were considered premenopausal if they were younger than 50 years and had levels of both gonadotrophins in the premenopausal range ($<40$ IU $l^{-1}$). Metastases occupying more than a third of the liver, lung lymphangitic metastases or brain dissemination were considered exclusion criteria. Patients with pleural effusion or bone blastic metastases as the only manifestation of disease were

Correspondence: E Bajetta
The following investigators should also be considered co-authors of this paper: E Arnoldi, Ospedale Civile, Seriate; S Barni, Ospedale S. Gerardo, Monza; A Fedeli, Ospedali Riuniti, Pesaro; A Jirillo, Ospedale Civile, Legnago, Italy
Received 15 August 1994; revised 19 December 1994; accepted 21 December 1994

excluded, as were those on concomitant anti-cancer therapies, other than limited radiotherapy fields on unevaluable bone lytic painful sites. Informed consent was obtained from all subjects before starting therapy, and the study was approved by the bioethics committee. Upon entry, patients were evaluated for disease extension by means of clinical examination, chest and skeletal radiographs, liver ultrasound or computerised tomographic scans, whole blood cell counts and blood chemistry.

### *Dose and schedule*

A depot formulation of goserelin 3.6 mg was administered by subcutaneous implantation in the abdominal wall every 4 weeks in an out-patient setting. Tamoxifen was self-administered at a total daily dose of 20 mg. At the time of each clinical examination, the physician questioned the patients directly in order to check that they were taking the drug regularly. In patient with a normal menstrual cycle, treatment was started in the early follicular phase. The treatment was continued until there were no signs of disease progression, providing no severe adverse effects appeared.

### *Response assessment*

The initial assessment of response was after 2 months of treatment. Clinical examinations were repeated monthly; the other investigations after 2 months of therapy and subsequently at 3 monthly intervals. If the treatment was stopped for any reason during the first 2 months of therapy, the patient was considered as a treatment failure but evaluable in terms of tumour response.

Response was assessed according to UICC criteria (Hayward *et al.*, 1977), an objective response being defined as either complete (CR) or partial remission (PR). In particular, CR for bone disease was defined as the disappearance of lytic metastases; partial recalcification was considered a PR. In no case was pain relief considered an objective response.

In the case of treatment discontinuation, patients were followed every 2 months in order to record their survival time. Toxicity was evaluated at each visit according to WHO (1979) criteria.

### *Endocrine investigations*

Blood samples for hormonal analysis were taken at baseline (pretreatment) and 2, 6 and 12 months after starting therapy. The samples were collected at the same time of day for each patient throughout the study (between 9.00 and 11.00 h) and the serum was separated and stored at $-20^{\circ}C$ until assay. Oestradiol ($E_2$) was measured using a radioimmunoassay kit obtained from Diagnostic Products (Los Angeles, CA, USA), which had a sensitivity of 5.5 pmol $l^{-1}$ and intra- and inter-assay coefficients of variation of 7% and 8.9% respectively. Follicle-stimulating hormone (FSH), luteinising hormone (LH) and testosterone (T) levels were determined as previously described (Bajetta *et al.*, 1994*a*).

For each hormone, all of the samples from the same patient were analysed in the same assay batch. All endocrine evaluations were performed at the Laboratory of Endocrinology of Milan's Istituto Nazionale Tumori.

### *Analysis of results*

The study was planned according to the Simon's optimal two-stage design (Simon, 1989), in order to test the hypothesis that the true response probability was less than 40% against the alternative hypothesis of a response probability of more than 60%. With type I and II error probability levels of 5% and 10% respectively, this design implies that the treatment must be rejected if fewer than 11 responses are observed at the end of the first stage (25 patients) or fewer than 32 at the end of the second stage (total of 66 patients).

The response duration was calculated from the onset of disease regression to the time of progression or last follow-up visit. Survival curves for the times to death and treatment failure (TTF) were obtained using the Kaplan–Meier method. TTF was calculated as the time elapsing from the start of treatment to the date of treatment discontinuation for any reason.

Quantitative endocrine data are reported as means ± s.e.m. The values of each analyte at baseline and at the time of the last available sampling were compared using the Wilcoxon signed-rank test. The adopted significance level was 5%.

### Results

### *Clinical results*

Sixty-four premenopausal women with previously untreated advanced breast cancer entered this trial between January 1991 and March 1993. Although two subjects were withdrawn early after refusing the second injection of goserelin, all of the 64 women enrolled were considered evaluable according to the intention to treat principle. The main patient characteristics of the enrolled patients are summarised in Table I.

After a median treatment duration of 11 months (range 1–39), objective responses included seven CRs (11%) and 19 PRs (30%), the overall response rate being 41% (95% confidence interval 28–52%). In women with a normal menstrual cycle, delayed amenorrhoea beyond the second month of therapy was observed in only one subject, in whom three goserelin administrations were required to induce amenorrhoea.

The median age on entry of the responding patients was 41 years (range 29–52), and all but two had a performance status ⩽ 1 (ECOG scale). With the exception of two patients in CR with spontaneous amenorrhoea lasting less than 1 year, all of the responders were actively menstruating at the start of therapy. With regard to the hormone receptor status of the primary or recurrent tumour, 20 responsive patients (77%) had both ER- and PgR-positive tumours; one patient in CR and three in PR were only ER positive, and a further two patients in PR had an unknown receptor status.

Sixteen of the 26 responders (62%) had a DFI ⩾ 2 years. Of the women who experienced an objective response, 15 (58%) had previously received adjuvant chemotherapy.

**Table I** Main patient characteristics

| *Characteristic* | *Number* |
|---|---|
| Entered/evaluable | 64/64 |
| Median age (range) | 43 (29–52) |
| ECOG performance status | |
| ⩽1/2 | 59/5 |
| DFI (years) | |
| <2/⩾2 | 18/46 |
| Receptor status | |
| ER positive | 52 (81%) |
| ER unknown | 12 |
| PgR positive | 42 (66%) |
| PgR negative | 11 |
| PgR unknown | 11 |
| Menstrual status | |
| Regular menses | 54 (84%) |
| Spontaneous amenorrhoea | 6 |
| Drug-induced amenorrhoea | 4 |
| Dominant disease | |
| Soft tissues | 35 |
| Viscera | 16 |
| Bone | 35 |
| Number of disease sites | |
| 1 | 44 (69%) |
| ⩾2 | 20 (31%) |
| Previous adjuvant chemotherapy | 32 (50%) |

When analysing the response rate by disease location, the vast majority of objective remissions occurred in soft tissues (14 CRs + four PRs; overall response rate 51%). It is worth pointing out that all of the patients achieving CR had only soft-tissue metastases, suggesting that the benefits of therapy were greater in the patients with a better prognosis. Only three tumour regressions (one CR + one PR on lung and one CR on pleura; overall response rate 19%) were observed in visceral locations, with none of seven liver metastatic sites achieving an objective response. The bone lytic metastases showed only partial recalcification in 37% of the cases. An objective response was documented in six patients with more than one disease location.

The median time to response was 4 months (range 2–17); the median response duration was 24 months (range 7–37 +) in the case of CR and 12 months (range 6–30 +) for PR (13 months for CR + PR). At the time of this analysis, nine patients (two in CR and seven in PR) were still continuing treatment. The TTF and survival curves are shown in Figures 1 and 2.

Compliance with treatment was highly satisfactory, and both drugs were administered regularly as scheduled in all patients. In the 62 patients evaluable for toxicity, the combination was extremely well tolerated; the main side-effect was hot flushes, which occurred in 74% of patients but were described as severe by only two women. A reduction in libido was reported by 26% of patients. No patient was withdrawn from the study because of pharmacological side-effects.

*Endocrine results*

Data on hormone levels were missing for many patients because blood samples were not available for logistic reasons. Therefore the endocrine effects of the association during the first 12 months of treatment were assessed in only 34 women.

**Figure 1** Time to treatment failure ($n$ = patients at risk).

**Figure 2** Overall survival ($n$ = patients at risk).

Serum LH fell below the sensitivity of the method used (0.5 IU $l^{-1}$) in all patients from month 2 onwards. Pretreatment FSH levels [25.59 ± 4.32 (mean ± s.e.m.) IU $l^{-1}$] were also decreased significantly ($P$ = 0.0001) during therapy, falling to 2.64 ± 0.19 after 8 weeks, and remaining unchanged subsequently (2.32 ± 0.16 IU $l^{-1}$ and 2.21 ± 0.22 IU $l^{-1}$ at 6 and 12 months respectively).

The combination induced a persistent suppression of $E_2$ levels within the range of values observed in castrated or post-menopausal women (<70 pmol $l^{-1}$) in all patients. Basal oestrogen levels (191.06 ± 62.29 pmol $l^{-1}$) were significantly decreased by an average of 92% over a 12 month period ($P$ = 0.0001).

Serum T levels did not appear to be affected by treatment ($P$ = 0.10), mean androgen value being 1.14 ± 0.17 nmol $l^{-1}$ after 12 months compared with 1.19 ± 0.10 nmol $l^{-1}$ at starting therapy.

### Discussion

Convincing evidence exists that LH–RH analogue goserelin is effective in premenopausal patients with advanced breast cancer (Blamey *et al.*, 1992), but castration levels of $E_2$ are only reached after 3–4 weeks compared with 2–7 days after surgical oophorectomy (Beksac *et al.*, 1983). Furthermore, treatment with the analogue makes menstruating patients post-menopausal without interfering with androgen precursor aromatisation in peripheral tissues, a process believed to be the major source of circulating oestrogens in post-menopausal woman (Santen, 1990).

The role of tamoxifen in the management of premenopausal breast cancer does not appear to be as well established (Sunderland and Osborne, 1991), although the increased oestrogen levels reported in young patients on tamoxifen therapy seem to imply a direct gonadal effect of the drug (Manni and Pearson, 1980). This ovarian stimulation could theoretically reverse the inhibitory effect of tamoxifen on the growth of breast cancer cells, and thus decrease anti-tumour activity.

In the present study, the combination of goserelin and tamoxifen therapy led to a remission rate of 41%, the median duration of response being 13 months. Although our clinical results in terms of remission rate and response duration are similar to those reported for goserelin alone (response rates ranging from 33% to 45% and median response durations ranging from 8 to 15 months; Dixon *et al.*, 1990; Kaufmann *et al.*, 1991; Blamey *et al.*, 1992; Bajetta *et al.*, 1994*b*), it remains an open question whether tamoxifen may offer any advantage for patients treated with medical castration by the analogue. No prospective study assessing the efficacy of the combination has yet been published although, in a retrospective study of 50 patients receiving the combination, an overall response rate of only 18% was reported; a further 30% of patients showed stable disease, but no significant survival difference between these two response groups was observed (Dixon *et al.*, 1991). Recently, updating the results of a large ongoing randomised trial comparing goserelin with goserelin plus tamoxifen therapy, Jonat *et al.* (1994) have reported that after a median follow-up of 23 months only the time to first progression was significantly longer in the combination group (7 *vs* 6 months), and that there was no significant difference in either the response rate or survival.

No endocrine antagonistic interaction between goserelin and tamoxifen was observed in our study, in agreement with previously reported data (Walker *et al.*, 1989). No patient actively menstruating at the start of therapy continued to have normal cycles or experienced a return to menses on treatment, and so the combination led to a profound suppression of peripheral gonadotrophin and $E_2$ concentrations (such hypogonadotrophic inhibition of oestrogen ovarian production being persistent over time). Furthermore, serum FSH values did not show any tendency to increase on long-term therapy, as has been observed with goserelin alone (Bajetta *et al.*, 1994*a*), the association proving to be more effective in suppressing serum levels of this hormone. It must be emphasised that FSH stimulates premenopausal oestrogen synthesis by increasing the generation of molecules of the aromatase enzyme in ovarian granulosa cells (Santen, 1990).

In conclusion, in this prospective trial, the association of goserelin and tamoxifen proved to be a feasible treatment: it was effective in terms of the endocrine changes induced in the host and was not associated with any increased toxicity. Although our clinical data show that the concurrent use of goserelin and tamoxifen does not appear to provide better results than the analogue alone in the palliation of advanced breast cancer in premenopausal patients, it is necessary to await publication of the definitive results of large well-controlled randomised studies before it can be reliably concluded that goserelin and goserelin plus tamoxifen are equivalent therapies, or whether the association may lead to an improvement in survival, which is a more important indicator of true patient benefit. This latter aspect would also be more relevant in view of the renewed interest in ovarian ablation as adjuvant treatment in primary breast cancer, although a higher risk of endometrial cancer has also been reported in tamoxifen users (van Leeuwen *et al.*, 1994).

### References

BAJETTA E, ZILEMBO N, BUZZONI R, CELIO L, ZAMPINO MG, COLLEONI M, ORIANA S, ATTILI A, SACCHINI V AND MARTINETTI A. (1994*a*). Goserelin in premenopausal advanced breast cancer: clinical and endocrine evaluation of responsive patients. *Oncology*, **51**, 262–269.

BAJETTA E, CELIO L, ZILEMBO N, BONO A, GALLUZZO D, ZAMPINO MG, LONGHI A, FERRARI L AND BUZZONI R. (1994*b*). Ovarian function suppression with the gonadotrophin releasing hormone (GnRH) analogue goserelin in premenopausal advanced breast cancer. *Tumori*, **80**, 28–32.

BEKSAC MS, KISNISCI HA, CAKAR AN AND BEKSAC M. (1983). The endocrinological evaluation of bilateral and unilateral oophorectomy in premenopausal women. *Int. J. Fertil.*, **28**, 219.

BLAMEY RW, JONAT W, KAUFMANN M, BIANCO AR AND NAMER M. (1992). Goserelin depot in the treatment of premenopausal advanced breast cancer. *Eur. J. Cancer*, **28**, 810–814.

BUCHANAN RB, BLAMEY RW, DURRANT KR, HOWELL A, PATERSON AG, PREECE PE, SMITH DC, WILLIAMS CJ AND WILSON RG. (1986). A randomized comparison of tamoxifen with surgical oophorectomy in premenopausal patients with advanced breast cancer. *J. Clin. Oncol.*, **4**, 1326–1330.

DIXON AR, ROBERTSON JFR, JACKSON L, NICHOLSON RI, WALKER KJ AND BLAMEY RW. (1990). Goserelin (Zoladex) in premenopausal advanced breast cancer: duration of response and survival. *Br. J. Cancer*, **62**, 868–870.

DIXON AR, JACKSON L, ROBERTSON JFR, NICHOLSON RI AND BLAMEY RW. (1991). Combined goserelin and tamoxifen in premenopausal advanced breast cancer: duration of response and survival. *Eur. J. Cancer*, **27**, 806–807.

HARVEY HA, LIPTON A, MAX DT, PEARLMAN HG, DIAZ-PERCHES R AND DE LA GARZA J. (1985). Medical castration produced by the GnRH analogue leuprolide to treat metastatic breast cancer. *J. Clin. Oncol.*, **3**, 1068–1072.

HAYWARD JL, RUBENS RD, CARBONE PP, HEUSON JC, KUMAOKA S AND SEGALOFF A. (1977). Assessment of response to therapy in advanced breast cancer. *Br. J. Cancer*, **35**, 292–298.

JONAT W, KAUFMANN M, BLAMEY RW, HOWELL A, COLLINS JP, COATES A, EIERMANN W, JAENICKE F, NJORDENSKOLD B, FORBES J AND KOLVENBAG G. ON BEHALF OF THE ZOLADEX TRIALIST GROUP. (1994). A randomised trial comparing Zoladex (goserelin) with Zoladex plus Nolvadex (tamoxifen) as first line treatment for premenopausal advanced breast cancer. *Proc. Am. Soc. Clin. Oncol.*, **13**, 58.

KAUFMANN M, JONAT W, SCHACHNER-WUNSCHMANN E, BASTERT G, MAASS H. ON BEHALF OF THE COOPERATIVE GERMAN ZOLADEX STUDY GROUPS. (1991). The depot GnRH analogue goserelin in the treatment of premenopausal patients with metastatic breast cancer – a five-year experience and further endocrine therapies. *Onkologie*, **22**, 23–29.

KLIJN JGM AND DE JONG FH. (1982). Treatment with a luteinizing hormone-releasing analogue (Buserelin) in premenopausal patients with metastatic breast cancer. *Lancet*, **i**, 1213–1216.

KLIJN JGM AND DE JONG FH. (1984). Long-term treatment with the LH RH-agonist buserelin (HOE 766) for metastatic breast cancer in single and combined drug regimens. In *LH-RH and its Analogues*, LaBrie F, Belanger A and Dupont A. (eds) pp. 425–437. Elsevier: Amsterdam.

MANNI A AND PEARSON OH. (1980). Antiestrogen-induced remissions in premenopausal women with stage IV breast cancer: effects on ovarian function. *Cancer Treat. Rep.*, **64**, 779–785.

MATTA WHM, SHAW RW AND BURFORD GD. (1988). Endocrinologic and clinical evaluation following a single administration of a gonadotrophin-releasing hormone agonist (Zoladex), in a depot formulation, to premenopausal women. *Fertil. Steril.*, **49**, 163–165.

RIBEIRO G AND SWINDELL R. (1988). The Christie Hospital adjuvant tamoxifen trial – status at 10 years. *Br. J. Cancer*, **57**, 601–603.

SANTEN RJ. (1990). Clinical use of aromatase inhibitors: current data and future perspectives. *J. Enzyme Inhibit.*, **4**, 79–99.

SANTEN RJ, MANNI A AND HARVEY H. (1986). Gonadotropin releasing hormone (GnRH) analogs for the treatment of breast cancer and prostatic carcinoma. *Breast Cancer Res. Treat.*, **7**, 129–145.

SIMON R. (1989). Optimal two-stage designs for phase II clinical trials. *Controlled Clin. Trials*, **10**, 1–10.

SUNDERLAND MC AND OSBORNE CK. (1991). Tamoxifen in premenopausal patients with metastatic breast cancer: a review. *J. Clin. Oncol.*, **9**, 1283–1297.

VAN LEEUWEN FE, BENRAADT J, COEBERGH JWW, KIEMENEY LALM, GIMBRERE CHF, OTTER R, SCHOUTEN LJ, DAMHUIS RAM, BONTENBAL M, DIEPENHORST FW, VAN DEN BELT-DUSEBOUT AW AND VAN TINTEREN H. (1994). Risk of endometrial cancer after tamoxifen treatment of breast cancer. *Lancet*, **343**, 448–452.

WALKER KJ, WALKER RF, TURKES A, ROBERTSON JFR, BLAMEY RW, GRIFFITHS K AND NICHOLSON RI. (1989). Endocrine effects of combination antioestrogen and LH-RH agonist therapy in premenopausal patients with advanced breast cancer. *Eur. J. Cancer Clin. Oncol.*, **25**, 651–654.

WHO. (1979). *WHO Handbook for Reporting Results of Cancer Treatment*, World Health Organization: Geneva.